# Community health workers and *promotores de salud*: a tiered framework for infectious disease prevention and care among California agricultural workers

**DOI:** 10.3389/fpubh.2026.1972841

**Published:** 2026-10-02

**Authors:** Kaivalya Kasturi, Nancy Burke, Geetha Sivasubramanian

**Affiliations:** 1 Clovis North High School, Clovis, CA, United States; 2 Department of Public Health, University of California, Merced, Merced, CA, United States; 3 Division of Infectious Diseases, Department of Medicine, University of California, San Francisco, Fresno, CA, United States

**Keywords:** agricultural workers, community health workers, farmworkers, infectious diseases, *promotores de salud*

## Abstract

Agricultural workers in the United States face a disproportionate burden of infectious diseases, including tuberculosis, HIV, coccidioidomycosis, viral hepatitis, and COVID-19, driven by occupational exposures, crowded housing, poverty, and limited healthcare access. Despite this burden, farmworkers have among the lowest rates of healthcare utilization of any US working population, and traditional clinic-based delivery models are poorly suited to the realities of agricultural labor. Community health workers (CHWs), known as promotores de salud in Latino communities, offer a well-established strategy for bridging this gap through shared language, culture, and lived experience with the communities they serve. This mini-review synthesizes evidence on CHW and promotora interventions for infectious disease prevention and care among agricultural workers, with particular attention to California's farmworker communities. Available evidence supports CHW roles in COVID-19 vaccination and outreach, tuberculosis treatment adherence through community-based directly observed therapy, HIV prevention and pre-exposure prophylaxis (PrEP) navigation, and hepatitis screening and linkage to care. However, existing programs have largely operated in isolation, and, to our knowledge, no CHW intervention for coccidioidomycosis has been developed despite its importance as an occupational infectious disease in California. To address this fragmentation, we propose a three-tiered framework that extends CHW roles from community-based prevention and early recognition to screening, linkage, and treatment support, and ultimately to formal integration with healthcare and public health systems. Implementing this framework will require infectious disease-specific training in the languages of the communities served, sustainable financing, and rigorous evaluation of an integrated cross-disease CHW model in California's agricultural communities.

## Introduction

Agricultural workers are essential to the United States food supply, with over 2.1 million hired farm workers employed nationally (1). California leads all states by a wide margin, employing over 370,000 hired workers, approximately 17% of the national total, more than three times the next largest state, Texas, at 5.5% (1). The Central Valley, Salinas Valley, and Eastern Coachella Valley are among the most productive agricultural regions in the state. Yet the workforce sustaining this production faces profound structural disadvantages. These workers are largely Latino immigrants, with approximately half lacking work authorization (2). Many speak Spanish as a primary language, and an estimated 9–16% speak Indigenous languages such as Mixteco, Zapoteco, or Triqui as their first language (3). Of the over 1,000 farmworkers participating in the CHAMACOS-Project-19 study in Monterey County, for example, 83% were born in Mexico, 10% spoke an Indigenous language, and 44% had a primary school education or lower (4). In addition, many suffer economic hardship: 80% of migratory and seasonal agricultural workers earn family incomes below the federal poverty level, and over half lack health insurance (5). In Sonoma County, only 30% of farmworkers had health insurance, and farmworkers were three times more likely to report poor health (6).

In addition to these structural disadvantages, agricultural workers face occupational hazards including musculoskeletal injuries, pesticide exposure, and heat-related illness (3). Crowded housing, workforce mobility, and limited sanitation further compound risks; 36% of farmworkers live with more than two people per bedroom, thereby facilitating the transmission of infectious diseases (2). Infectious diseases represent a particularly significant and often underrecognized burden in this population. Tuberculosis prevalence among US crop workers has shown an increasing trend, with agricultural activity associated with increased tuberculosis burden in the context of crowded housing, demographic factors, and limited healthcare access (7). HIV and sexually transmitted infection (STI) risk is elevated among Latino migrant farmworkers, driven by social isolation, limited healthcare access, and cultural stigma around testing (8). In California, coccidioidomycosis (Valley Fever)^1^ incidence increased nearly 800% between 2000 and 2018, with the highest rates in the agriculturally intensive San Joaquin Valley. Among Hispanic farmworkers, dust exposure and work with root and bulb vegetable crops have been identified as occupational risk factors (9, 10). During the COVID-19 pandemic, SARS-CoV-2 seroprevalence among farmworkers in Monterey County reached 19%, with residential crowding and workplace exposures independently associated with infection risk (4). Viral hepatitis, particularly hepatitis B and C, adds further burden among migrant populations with limited screening infrastructure (11).

Despite this disproportionate disease burden, farmworkers have among the lowest rates of healthcare utilization of any working population in the United States. In a national survey, only 55% reported any healthcare visit in the preceding 2 years (12). Immigration status, workforce mobility, and lack of insurance fragment continuity of care and limit access to preventive services (13), and California farmworkers, in particular, cite cost and distance as primary reasons for not seeking care (14). While healthcare outreach services are increasingly offered in Spanish, the estimated 10% of farmworkers who speak Indigenous languages face a barrier within a barrier, as health services in these languages remain extremely limited (3). Traditional clinic-based delivery models require farmworkers to travel to fixed facilities during business hours, a model that is fundamentally mismatched with the realities of agricultural labor (15). Cultural and linguistic barriers, geographic isolation, and fear of deportation further limit engagement with formal healthcare systems (16). The convergence of poverty, occupational exposures, crowded living conditions, and limited healthcare access creates a syndemic vulnerability that concentrates infectious disease risk in this population (17). Bridging this gap requires a workforce model rooted in the communities themselves.

Community health workers (CHWs), known as *promotores de salud* in Latino communities, are frontline public health workers who share language, culture, and lived experience with the communities they serve (18, 19). Systematic reviews have confirmed that CHW interventions improve chronic disease management, increase access to preventive care, and reduce healthcare costs among underserved populations (18, 20), and CHWs have demonstrated effectiveness both in settings with limited health workforce resources and in settings where health disparities persist despite well-developed health systems (19). For agricultural worker communities, where the barriers described above converge, CHWs can deliver health education and screening at worksites and housing camps, provide services in Spanish and Indigenous languages, navigate patients through unfamiliar health systems, support treatment adherence across moves between regions, and build trust where fear of institutional contact limits engagement with care. To our knowledge, no review has synthesized the evidence specifically examining CHW and promotora interventions for infectious disease prevention and care among agricultural workers. This mini-review addresses that gap by examining the role of CHWs in infectious disease efforts relevant to California’s farmworker communities, including COVID-19, tuberculosis, HIV, hepatitis, and coccidioidomycosis. It further considers implementation factors, workforce development, and policy considerations necessary to sustain and expand these programs.

## CHWs and *promotores de salud*: history and roles

Community health workers (CHWs) are health workers who typically operate in community settings rather than fixed health facilities, with training that is usually role and community-specific rather than based on a licensed clinical profession (18, 19). Compared with other members of the health workforce, they have an unusually close understanding of the communities they serve, and are known by many titles, including *promotores de salud*, lay health advisors, and patient navigators^2^ (18, 19). Modern CHW models are often traced to China’s barefoot doctor program of the 1950s. Similar programs in Latin America, South Asia, and Sub-Saharan Africa shaped the global emphasis on community-based primary healthcare, formalized in the 1978 Alma-Ata Declaration (19). In the United States, early models included the Indian Health Service’s Community Health Representative program and community outreach workers in federally funded neighborhood health centers during the War on Poverty era (18). The *promotora de salud* model has deep roots in US-Mexico border communities, where Spanish-speaking lay health workers have provided culturally grounded outreach to underserved Latino populations for decades (21). Following expansion in the 1970s and contraction during the 1980s, CHW and promotora programs have experienced marked growth over the past two decades (19).

A pivotal milestone came in 2009, when the US Bureau of Labor Statistics formally recognized community health workers as a distinct occupational category (22). The 2010 Patient Protection and Affordable Care Act (ACA) further advanced CHW integration by recognizing CHWs as healthcare workforce members and promoting care coordination and interdisciplinary teams (23). The ACA also provided impetus for state Medicaid agencies to integrate CHW services (24), and the concurrent shift toward value-based payment models increased health system interest in CHWs as a strategy for addressing social needs and reducing preventable utilization among patients with complex needs (18). As of 2025, 29 states allowed Medicaid reimbursement through state plan amendments, 1,115 waivers, managed care organizations, or combinations of these approaches (25). The Community Health Worker Core Consensus (C3) Project established standardized roles and competencies across work settings, including cultural mediation, health education, care coordination, outreach, advocacy, and capacity building (22, 26). In practice, CHW functions span clinical support such as screenings and health assessments, connections to community resources including housing and transportation, health education and behavioral coaching, and direct tangible assistance that addresses the material conditions underlying health disparities (21, 27).

Evidence for CHW effectiveness has grown substantially, with randomized trials and systematic reviews demonstrating improvements in components of the “triple aim” of healthcare: population health, patient experience, and costs (18). Disease-specific reviews have shown CHW-led improvements in chronic disease management, HIV antiretroviral adherence, preventive service uptake, including cancer screening, and primary care access among underserved populations (18, 27, 28). Some programs have generated net savings, with one study of Arkansas Medicaid beneficiaries demonstrating a 23.8% reduction in annual spending compared with propensity score-matched controls (18). CHWs also advance health equity by addressing social needs and advocating for systems and policy change at individual, institutional, and community levels (18).

## CHW and promotora interventions for infectious diseases in farmworker populations

We conducted a narrative literature search of PubMed and Google Scholar for articles published through August 2026, using combinations of terms including *community health worker*, *promotora de salud*, *lay health worker*, *farmworker*, *agricultural worker*, *migrant*, and *underserved*, combined with terms for infectious diseases relevant to agricultural worker communities. Additional articles were identified through reference lists of relevant publications. We prioritized studies describing CHW or promotora-delivered interventions addressing infectious disease prevention, screening, linkage to care, or treatment support among agricultural workers. Where farmworker-specific evidence was limited, studies in analogous underserved or hard-to-reach populations were included when they addressed similar barriers to infectious disease prevention or care. Commentaries, opinion pieces, and reports without a described intervention were not included as intervention evidence. Original source publications were reviewed for study population, intervention characteristics, outcomes, and reported limitations. Because this was a narrative mini-review rather than a systematic review, no formal risk-of-bias assessment was performed, and the search was not intended to be exhaustive.

Table 1 summarizes the available evidence on CHW interventions for infectious diseases relevant to farmworker communities. Across diseases, CHW programs have targeted a consistent set of barriers: cultural and linguistic isolation, stigma around testing and diagnosis, limited health literacy, distrust of formal healthcare systems, and the practical challenges of accessing care during agricultural work seasons. The strategies employed are also similar across pathogens, including culturally tailored education delivered by trusted community members, navigation through screening and referral pathways, accompaniment to clinical appointments, and support for treatment adherence.

**Table 1:** Community health worker (CHW) and promotora interventions for infectious diseases in farmworker populations.

Infectious disease	Key barriers	CHW intervention	Setting/study design	Key findings	Limitations	References
COVID-19	Crowded housing, vaccine hesitancy, time poverty, and limited healthcare access	1. ¡Ándale! ¿Qué Esperas?: trusted messenger, multichannel outreach, vaccine navigation 2. Cosecha network: multi-organization promotor network, vaccination navigation 3. CHW outreach and contact tracing	1. Latino communities, California; program/campaign evaluation with regression analysis 2. Farmworker corridor, Florida; program/network field report 3. Rural farmworkers, North Carolina; qualitative evaluation of program adaptations	1. Increased vaccine uptake, reduced hesitancy. 2. Improved access to services, built a statewide promotor network across Florida. 3. Addressing mental health needs, helping with technology, and making vaccines more accessible	Short-term pandemic outcomes only; long-term sustainability unknown	(29–32)
Tuberculosis (TB)	Overcrowded housing, limited healthcare access, and mobility-disrupting long treatment courses	1. Nurse/CHW-led LTBI care 2. CHW house-to-house case finding + treatment supervision 3. CHW active case finding + treatment support 4. CHW screening + contact tracing	1. Homeless, Los Angeles; single-arm pilot (*N* = 50) vs. historical clinic control 2. Rural Ethiopia; before/after with control-zone comparison 3. Rural India; quasi-experimental operational research (intervention vs. control) 4. Rural Mozambique; before/after, intervention vs. control districts	1. 92% LTBI completion vs. 65% 2. Notifications doubled, success 77% → 93% 3. Notifications 46 → 106/100,000 4. Notifications +14.6% vs. − 16.5%	No CHW TB intervention tested in the US/California farmworkers; evidence from analogous hard-to-reach populations	(33–36)
HIV/sexually transmitted infections	Social isolation, limited healthcare access, cultural stigma around contraceptives, testing, and treatment	1. Lay health adviser (education, stigma reduction, HIV testing) 2. Progreso en Salud (social network HIV risk reduction) 3. PROGRESO-II (PrEP navigation) 4. SEPA (HIV prevention, communication, condom use)	Underserved Latinos, Southwestern US; pre/post evaluation (*N* = 579) 2. Latino seasonal farmworkers, Florida; pilot pre/post (brief report, *N* = 114) 3. Latino seasonal farmworkers, Florida; formative, mixed-methods materials development (*N* = 16) 4. Latino immigrants, Florida; efficacy study, pre/post cohort (*N* = 234)	1. Reduced HIV/AIDS stigma and increased HIV knowledge 2. Increased HIV prevention knowledge and condom use 3. Increased PrEP awareness and acceptability 4. Increased condom use, communication, and HIV knowledge	There is little evidence on whether these changes resulted in a sustained reduction in HIV incidence among the communities.	(37–40)
Viral hepatitis	Limited screening infrastructure, low awareness, stigma	1. Hepatitis Outreach Network (screening, linkage to care) 2. CHW-led Hepatitis education (knowledge, testing)	Foreign-born immigrant communities, New York; screening program evaluation with linkage-to-care outcomes 2. Cambodian American men and women, Washington; RCT (sex-stratified analysis)	1. Increased screening and linkage to care 2. Increased Hepatitis B knowledge and self-reported testing	No CHW hepatitis intervention has been conducted in farmworker populations; existing evidence from other immigrant communities.	(41, 42)
Coccidioidomycosis	Low awareness (26.4% among Hispanic Californians), occupational dust exposure	None developed or tested	N/a	N/a	No CHW intervention exists currently	(43)

LTBI, Latent Tuberculosis Infection; SEPA, Salud, Educación, Prevención, y Autocuidado; PrEP, Pre-exposure prophylaxis.

Community health worker (CHW) and promotora interventions for infectious diseases relevant to agricultural worker populations, including COVID-19, tuberculosis, HIV and sexually transmitted infections, viral hepatitis, and coccidioidomycosis, summarizing key barriers, intervention strategies, settings, key findings, and limitations across studies conducted in farmworker and analogous hard-to-reach populations.

The strongest evidence exists for COVID-19 vaccination and outreach, where CHW and promotora-led campaigns in California, Florida, and North Carolina increased vaccine uptake, reduced hesitancy, and expanded access to services among farmworker communities (29–32). For tuberculosis, CHW-led case finding, screening, and treatment support have improved detection and treatment completion among analogous hard-to-reach populations, although no such intervention has been tested specifically in US farmworkers (33–36). CHW and promotora-led HIV programs using education, stigma reduction, condom promotion, and pre-exposure prophylaxis (PrEP) navigation have shown improved knowledge, testing, and prevention behaviors, though sustained reductions in HIV incidence have not been demonstrated (37–40). Evidence for CHW-led hepatitis screening, education, and linkage to care is emerging but limited to non-farmworker immigrant communities (41, 42). Notably, no CHW intervention for coccidioidomycosis has been developed or tested in any population, despite its significance as an occupational infectious disease among California agricultural workers (43). Across these diseases, CHWs performed overlapping core functions: education, screening facilitation, and treatment support, yet each program was developed, funded, and staffed independently.

## Discussion

### A tiered framework for CHW roles in infectious disease

Rebuilding training, funding, and organizational infrastructure separately for each disease is neither efficient nor sustainable. To our knowledge, no framework has been proposed for deploying CHWs across infectious disease prevention and care for agricultural workers. We propose a three-tiered framework that organizes CHW roles according to increasing levels of clinical and public health integration, adaptable to local disease epidemiology and scalable as workforce capacity grows (Figure 1). The tiers reflect increasing organizational integration rather than a hierarchy of CHW value or importance.

**Figure 1:**
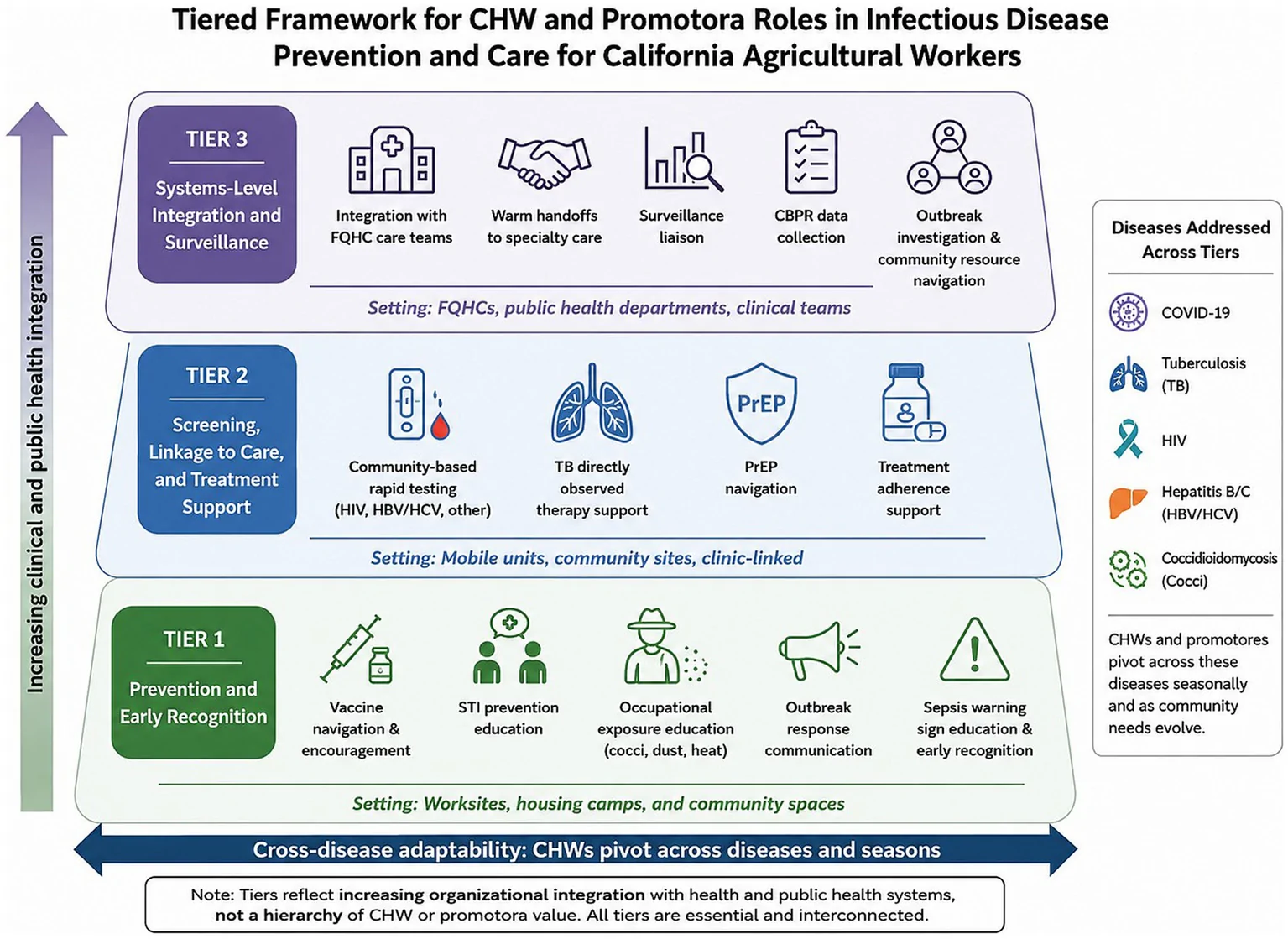
Three-tiered framework for community health worker (CHW) and promotora roles in infectious disease prevention and care for California agricultural workers. Tier 1 (Prevention and Early Recognition) comprises community-facing activities at worksites, housing camps, and community spaces, including vaccine navigation, sexually transmitted infection prevention education, occupational exposure education, outbreak response communication, and sepsis warning-sign recognition. Tier 2 (Screening, Linkage to Care, and Treatment Support) comprises community-based rapid testing, directly observed therapy support for tuberculosis, pre-exposure prophylaxis navigation, and treatment adherence support through mobile and clinic-linked units. Tier 3 (Systems-Level Integration and Surveillance) comprises integration with federally qualified health center care teams, warm handoffs to specialty care, surveillance liaison roles, community-based participatory research data collection, and outbreak investigation. Tiers reflect increasing organizational integration rather than a hierarchy of CHW or promotora value, and CHWs pivot across diseases and seasons.

### Tier 1: prevention and early recognition

This foundational tier comprises community-facing activities that do not require direct participation in clinical care. CHWs can deliver vaccine promotion and navigation for COVID-19, influenza, hepatitis B, and human papillomavirus (HPV), an area where farmworker-specific evidence already supports effectiveness (28). STI prevention education, including condom distribution and risk reduction counseling, has been demonstrated in farmworker populations (38, 44) and can be extended into routine programming. Occupational exposure education is a critical unmet need, particularly for coccidioidomycosis, where CHWs could deliver dust-exposure education, promote respiratory protection during soil-disrupting labor, and teach symptom recognition. However, no such program has been tested (43). During outbreaks, CHWs have demonstrated capacity for contact tracing, risk communication, and quarantine support (29). An aspirational extension would train CHWs to provide culturally adapted education on warning signs of severe infection requiring urgent evaluation, consistent with calls to integrate infection prevention with community-level sepsis awareness (45).

### Tier 2: screening, linkage to care, and treatment support

HIV, hepatitis B and C, tuberculosis, and coccidioidomycosis share a common challenge: diagnosis requires the patient to be in the healthcare system, and treatment requires sustained engagement over weeks to months, points at which mobile farmworkers are particularly vulnerable to being lost to follow-up. CHWs can navigate screening and confirmatory testing and support completion of prolonged treatment courses for tuberculosis, hepatitis C, and coccidioidomycosis. CHW-led directly observed therapy is both effective and substantially less costly than facility-based models (46). PrEP navigation for HIV-negative farmworkers at risk has shown feasibility in pilot programs (39) but requires expansion. Community-based rapid testing for HIV and hepatitis B/C using point-of-care tests with performance comparable to conventional laboratory assays (47) could be adapted for farmworker settings through mobile units at worksites and housing camps. Across these infections, CHWs address barriers including inflexible work schedules, lack of transportation, concerns about immigration status exposure, medication costs, and the need for accompaniment and interpretation at specialist visits (2).

### Tier 3: systems-level integration and surveillance

CHWs should be embedded within infectious disease infrastructure rather than operating as supplementary outreach workers. This tier requires formal organizational partnerships, defined referral and communication pathways, appropriate supervision, and integration with clinical or public health teams. California surveillance systems include tuberculosis, HIV, viral hepatitis, and coccidioidomycosis, and CHWs can serve as community liaisons to improve engagement with populations that formal systems struggle to reach. CHWs can participate in outbreak investigation teams, contribute epidemiologic data through community-based participatory research (CBPR) partnerships, and advocate for infectious disease resources, including mobile testing, treatment access, and isolation housing during outbreaks. Patient navigators already extend care beyond the clinic by connecting patients with community resources such as nutritional support, rent assistance, transportation, and housing. Integration into federally qualified health center (FQHC) infectious disease workflows would build on this foundation by embedding CHWs within clinical care teams, establishing formal referral and communication pathways, and facilitating warm handoffs between community outreach and specialty care (18).

Because the tiers are additive rather than disease-specific, a CHW trained through this framework can pivot from occupational exposure education during periods of high dust exposure to influenza vaccine navigation in fall and winter to tuberculosis screening support year-round, providing continuity that single-disease pilots cannot.

## Implementation considerations

This framework is a conceptual proposal. Whether organizing CHW infectious disease roles into progressive tiers is feasible, acceptable, and more effective than single-disease programs has not been tested. Pilot programs in regions where multiple infectious diseases converge in agricultural populations, such as California’s San Joaquin Valley, could evaluate whether CHWs trained across diseases provide more continuous support and better outcomes than current approaches. FQHCs and county public health departments are potential institutional homes for such pilots, with infectious disease clinicians and academic medical centers contributing clinical training content and evaluation expertise, and CHW organizations and farmworker communities shaping program design and delivery.

Training should increase with the level of responsibility expected at each tier. CHWs providing prevention and early recognition will need training in vaccine-preventable infections, STIs, tuberculosis, coccidioidomycosis, occupational exposure reduction, and recognition of symptoms that require medical evaluation. Those involved in screening, linkage to care, and treatment support will need additional training in testing pathways, confidentiality, referral procedures, medication adherence, and clinical escalation. Systems-level roles will require defined communication pathways, supervision, and training in outbreak response and community-engaged data collection and storage. Data integration remains an unresolved implementation challenge: patient navigators in other settings have collected valuable information about patients’ social and health needs, but this data is often stored in systems that do not communicate with the electronic health record (EHR), limiting its accessibility to clinical teams. As CHW roles expand into systems-level data collection, programs will need to establish mechanisms for integrating CHW-collected data into clinical and public health information systems while maintaining patient confidentiality and community trust. These modules should build on existing CHW certification curricula and be developed with input from CHWs, infectious disease clinicians, public health departments, and training programs. Training must be available in the languages used by farmworker communities, including Spanish and Indigenous languages such as Mixteco, Zapoteco, and Triqui, as nearly half of the CHW workforce provides services in a language other than English (48). CBPR partnerships can help ensure that curricula reflect the experiences and priorities of farmworker communities (49).

Financing remains a major barrier. As of 2025, 29 states, including California, allowed Medicaid reimbursement for CHW services (25), and California’s Advancing and Innovating Medi-Cal (CalAIM) initiative may provide a mechanism to support infectious disease-related CHW activities. Reimbursement models must account not only for individual encounters but also for outreach travel, training, supervision, interpretation, and coordination with clinical and public health teams. Current payment levels may not be sufficient: one modeling study estimated that approximately $53 per 30-min visit would be needed to cover program costs (50). Although the proportion of FQHCs employing CHWs increased from 20% in 2016 to 28% in 2018, financial barriers remain substantial (51). Additional support may be needed through county public health funding, Centers for Disease Control and Prevention (CDC) cooperative agreements, Health Resources and Services Administration (HRSA) grants, and employer-supported occupational health programs, with safeguards protecting CHW independence, worker confidentiality, and community trust.

## Gaps and priorities

California-specific data on CHW infectious disease interventions among farmworkers remain limited, with most evidence derived from other states and international settings. Cost-effectiveness data are particularly sparse. No CHW intervention for coccidioidomycosis has been developed or tested in any population, despite the concentration of disease and agricultural labor in the San Joaquin Valley (9, 43, 52). Indigenous-language-speaking farmworkers remain underserved even within Spanish language CHW programs, and addressing this gap will require dedicated recruitment and training of CHWs from these communities, rather than simply translating existing materials.

## Conclusion

CHWs and *promotores de salud* have supported infectious disease education, prevention, screening, linkage to care, and adherence to treatment among agricultural workers. However, most programs remain focused on a single disease and are supported through short-term funding. We propose a tiered framework that expands CHW roles from community-based prevention and early recognition to screening, treatment support, and integration with healthcare and public health systems. Central California provides an important setting to test this approach because of its large agricultural workforce, existing CHW infrastructure, and substantial burden of coccidioidomycosis and other infectious diseases. Further studies are needed to determine whether an integrated cross-disease CHW model is feasible, sustainable, and effective in improving infectious disease prevention and care among farmworker communities.

## Footnotes

1 Coccidioidomycosis, commonly known as Valley Fever, is a fungal infection caused by inhalation of *Coccidioides* spores that become airborne when soil is disturbed. It is endemic to arid regions of the southwestern United States, particularly California’s Central Valley. Most infections are asymptomatic or cause self-limited pneumonia, but a minority progress to severe disseminated disease. Agricultural workers are at elevated risk due to occupational dust exposure.

2 Patient navigators are typically based in fixed health facilities such as primary care clinics, oncology centers, or emergency departments, where they coordinate interdisciplinary care and connect patients with community resources. CHWs and promotores de salud, by contrast, operate primarily in community settings, though the roles may overlap.
